# Prediction of potential habitat of *Verbena officinalis* in China under climate change based on optimized MaxEnt model

**DOI:** 10.3389/fpls.2025.1563070

**Published:** 2025-03-19

**Authors:** Shimao Chen, Zixuan Jiang, Jia Song, Tao Xie, Yu Xue, Qingshan Yang

**Affiliations:** ^1^ College of Pharmacy, Anhui University of Chinese Medicine, Hefei, China; ^2^ Anhui Province Key Laboratory of Research & Development of Chinese Medicine, Hefei, China; ^3^ Institute of Conservation and Development of Traditional Chinese Medicine Resources, Anhui Academy of Chinese Medicine, Hefei, China

**Keywords:** *Verbena officinalis*, MaxEnt model, environmental variables, climate change, potential habitat prediction

## Abstract

*Verbena officinalis* is an important medicinal plant widely used in traditional Chinese medicine for the treatment of rheumatism, insomnia, and liver and gallbladder diseases. Its resources primarily rely on wild populations, which are insufficient to meet the increasing market demand. Furthermore, climate change exacerbates the uncertainty of its distribution range. This study employs an optimized MaxEnt model to predict the potential distribution of *V. officinalis* under current and future climate scenarios in China. Based on 445 effective occurrence records and 90 environmental variables (covering climatic, soil, and topographic factors), the study selected key variables influencing the distribution through correlation analysis and variable contribution rates, and optimized model parameters to improve prediction accuracy (AUC = 0.934). Results showed that, under current climate conditions, the total suitable habitat area of *V. officinalis* is 2.06 × 10^6^ km^2^, accounting for 21.39% of China’s land area, mainly distributed in central, eastern, and southern China. The minimum temperature of the coldest month (bio_6, contribution rate 72.8%) was identified as the key factor influencing distribution, while November precipitation (prec_11) and annual temperature range (bio_7) also played important roles. Under future climate change scenarios (SSP1-2.6 and SSP5-8.5), the total suitable habitat area shows an overall increasing trend, reaching a maximum in the 2070s under the high-emission scenario (an increase of 3.6 × 10^5^ km^2^ compared to the current distribution). Expansion was primarily observed in northern high-latitude regions. The geometric centroid of suitable areas demonstrated a significant northward shift, reflecting the adaptive expansion potential of *V. officinalis* in response to warming climates. This study highlights the significant impact of temperature and precipitation on the distribution of *V. officinalis* and provides scientific evidence for its conservation, cultivation planning, and sustainable development in the context of climate change.

## 1 Introduction

Global climate change has emerged as one of the most pressing environmental challenges of the 21st century, profoundly altering plant growth patterns, reproductive success, and biogeographic distributions (Parmesan and Yohe, 2003). Rising temperatures, shifting precipitation regimes, and increased frequency of extreme weather events, as highlighted in the latest IPCC report, are driving rapid ecological niche modifications and threatening biodiversity. To address these challenges, numerous ecological niche models (ENMs), such as DO-MAIN, GARP, GAM, GLM, ENFA, Bioclim, and MaxEnt, have become indispensable tools for predicting species’ potential distributions by correlating occurrence data with environmental variables (Warren et al., 2013 Hernandez-Lemus et al., 2014). Among ENMs, the Maximum Entropy Model (MaxEnt) has demonstrated superior accuracy in medicinal plant studies, particularly with limited sample sizes (Yi et al., 2018). Through comparative analysis, the MaxEnt model demonstrated superior predictive accuracy in independent regions, achieving higher mean sensitivity (0.84) and AUC values (e.g., 0.957 for *Asparagus asparagoides*), while effectively reducing overfitting through regularization (Shabani et al., 2016). It is capable of handling presence-only data and nonlinear relationships through feature regularization (Phillips et al., 2006). For instance, MaxEnt has been widely applied to studies on the growth zoning and responses to climate change of medicinal plants such as *Forsythia suspensa* (Wang et al., 2024), *Angelica dahurica* (Zhang et al., 2024), *Cirsium lineare* (Fang et al., 2024), and *Rubus idaeus* (Gao et al., 2024). This makes it highly suitable for modeling species like *Verbena officinalis*, which often exhibit fragmented occurrence records. However, existing research on medicinal plants largely focuses on a single climate scenario or default model parameters, neglecting parameter optimization and the integration of multiple factors. This study innovatively combines parameter adjustment using the Kuenm package with high-resolution soil, topography, and climate variables to predict the distribution of *Verbena officinalis*, addressing a critical gap in the utilization of temperate herbal resources under climate change.

*Verbena officinalis* L., a perennial herbaceous plant of the Verbenaceae family, is widely distributed in temperate regions. Revered as a “sacred herb” in European folk medicine since antiquity, it featured prominently in Druidic rituals and medieval Christian healing practices (De Natale and Pollio, 2007). In China, its medicinal use dates to the Han Dynasty (206 BCE–220 CE), documented in the *Shennong Bencao Jing* for treating inflammatory disorders and liver ailments. Modern pharmacological studies validate its therapeutic potential, identifying over 100 bioactive compounds, including iridoid glycosides with anti-tumor properties and phenylethanoid glycosides exhibiting neuroprotective effects (Dai et al., 2023). Since its official inclusion in the Pharmacopoeia of China in 1995, its medicinal value has received increasing attention. However, China’s reliance on wild populations—which constitute 60% of global *V. officinalis* resources—has created a critical sustainability challenge: domestic cultivation meets ≤15% of market demand, while climate-driven habitat fragmentation threatens traditional harvesting regions in Guizhou and Henan provinces (Kubica et al., 2020). Therefore, using the MaxEnt model to investigate the key environmental factors influencing the optimal growth of *V. officinalis* and their impact on its suitable habitat distribution is of great significance for the conservation of this plant resource, its cultivation, and its sustainable development in the future.

This study focuses on China as the research area, collecting and organizing distribution data based on field investigations and historical specimen records. Combined with environmental factors such as climate, topography, and soil, the MaxEnt model’s optimal parameters were set using the Kuenm package to simulate and predict the suitable habitat distribution of *V. officinalis* under current and future climate change scenarios. ArcGIS was utilized to analyze and visualize the changes in the size and core distribution patterns of suitable habitats, aiming to provide theoretical guidance for the sustainable development and artificial cultivation of *V. officinalis* resources.

## 2 Materials and methods

### 2.1 Collection and processing of distribution data for *V. officinalis*

**Figure 1:**
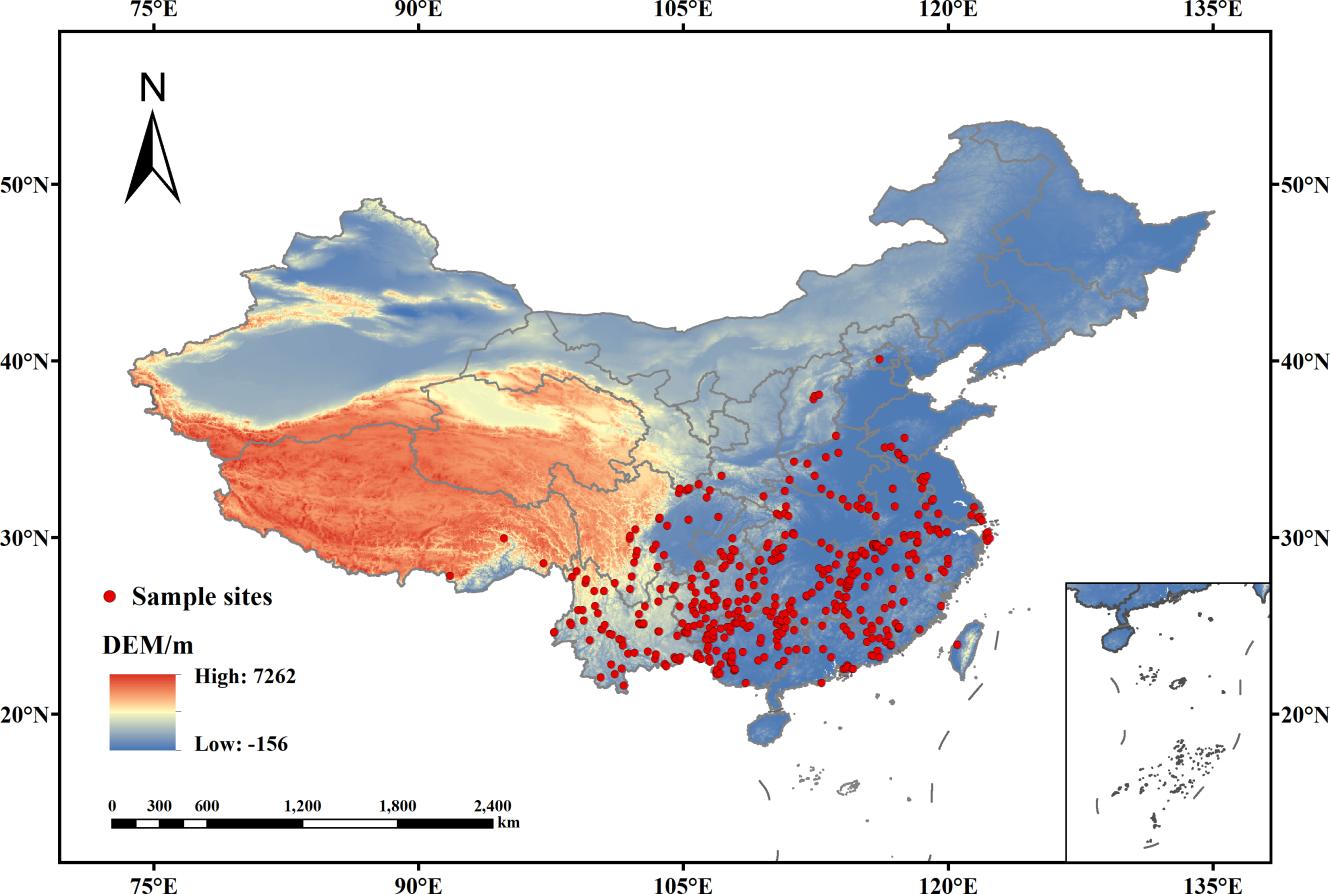
Spatial distribution of *V. officinalis* occurrence points in China.

The distribution data of *V. officinalis* were sourced from the National Specimen Information Infrastructure of China (NSII, http://www.nsii.org.cn/) and the China Virtual Herbarium (CVH, http://www.cvh.org.cn/). The study only collected distribution points of *V. officinalis* after 1980, and cross-checked these with Google Maps to supplement missing geographic coordinates. A total of 465 distribution points for *V. officinalis* were collected. To reduce spatial autocorrelation, each grid (5 km × 5 km) was used as a standard, and ENMTools was employed to prune redundant data points, ensuringthat each grid cell contains only one distribution point (Fu et al., 2024). Finally, 445 valid distribution points were obtained, and the specific distribution locations are shown in **Figure 1**.

### 2.2 Sources and processing of environmental data

The environmental data utilized in this study, encompassing both contemporary and future climate data, were sourced from the WorldClim database (https://worldclim.org/). This dataset includes 19 bioclimatic variables (Bio1–Bio19), as well as monthly precipitation, maximum and minimum temperatures, and mean temperatures for each of the 12 months. Three topographic factors—elevation, slope, and aspect—were extracted from Digital Elevation Model (DEM) data. Soil texture type data were obtained from the Harmonized World Soil Database (HWSD). Future climate projections are derived from the Coupled Model Intercomparison Project Phase 6 (CMIP6), specifically employing the Beijing Climate Center Climate System Model (BCC-CSM 2-MR) (Gao et al., 2023).In this study, we examined shared socioeconomic pathways (SSP) emission scenarios for the 2030s (average values from 2021 to 2040), 2050s (2041 to 2060), 2070s (2061 to 2080), and 2090s (2081 to 2100) under two extreme scenarios: SSP1-2.6 and SSP5-8.5. To ensure consistency, the spatial resolution of the 90 environmental variables was standardized to 2.5 arc minutes based on the bioclimatic data. Detailed information regarding the 90 environmental variables is presented in **Table 1**. Among them, prec 1-12, tavg 1-12, tmax 1-12, and tmin 1-12 each contain 12 variables, corresponding to the data for the 12 months.

**Table 1:** Detailed information on the 90 environmental variables.

Variable code	Environmental factor	Variable code	Environmental factor
prec 1-12	January to December precipitation	alt	Altitude
tavg 1-12	January to December average temperature	slope	Slope
tmax 1-12	January to December maximum temperature	aspect	Aspect
tmin 1-12	January to December minimum temperature	zbyl	Vegetation Classification
bio1	Annual Mean Temperature	coarse	Coarse fragments
bio2	Mean Diurnal Range	sand	Sand
bio3	Isothermality	slit	Slit
bio4	Temperature Seasonality	clay	Clay
bio5	Max Temperature of Warmest Month	bulk	Bulk Density
bio6	Min Temperature of Coldest Month	ref_bulk	Reference Bulk Density
bio7	Temperature Annual Range	org_cbn	Organic Carbon Content
bio8	Mean Temperature of Wettest Quarter	ph	pH in water
bio9	Mean Temperature of Driest Quarter	n	Total nitrogen content
bio10	Mean Temperature of Warmest Quarter	cn	Carbon/Nitrogen ratio (C/N)
bio11	Mean Temperature of Coldest Quarter	cec_soil	CEC soil
bio12	Annual Precipitation	cec_clay	CEC clay
bio13	Precipitation of Wettest Month	teb	TEB
bio14	Precipitation of Driest Month	bsat	Base Saturation
bio15	Precipitation Seasonality	alum_sat	Aluminium saturation
bio16	Precipitation of Wettest Quarter	esp	Exchangeable Sodium Percentage
bio17	Precipitation of Driest Quarter	eq	Calcium Carbonate
bio18	Precipitation of Warmest Quarter	gypsum	Gypsum content
bio19	Precipitation of Coldest Quarter	elec_con	Electric Conductivity

Due to the potential high correlation among environmental variables, key environmental variables for MaxEnt modeling were selected from the initial set of 90 variables through the following steps: (1) All 90 environmental variables were combined with 445 distribution points and run in MaxEnt to determine the contribution rate of each variable to the model; (2) The 90 environmental variables were imported into ENMTools software to calculate the Pearson correlation coefficients |r| between every pair of variables; (3) Variables with |r| < 0.8 were selected, and those with a contribution rate greater than or equal to 0.4 in the initial model were retained. Based on these criteria, a total of 13 environmental variables were ultimately retained for subsequent model development, optimization, and evaluation, namely bio_6, bio_7, zbyl, prec_09, slope, prec_11, alt, tmax_04, eq, bsat, alum_sat, aspect, and gypsum.

### 2.3 Establishment, optimization, and evaluation of the MaxEnt model

The processed *V. officinalis* distribution data was saved in CSV format and imported into MaxEnt along with the filtered environmental factors. The model parameters were set as follows: 25% of the distribution data was used as the test set (random test percentage), and 75% as the training set. The Bootstrap method was applied, with the default maximum background points set to 10,000, and the random seed was enabled to ensure reproducibility. The number of replicates was set to 10, and the output format was set to logistic values.

Additionally, considering that the predictive performance of the MaxEnt model is influenced by the regularization multiplier (RM), feature combination (FC), and Max number of background points, the parameters for the MaxEnt model were optimized using the Kuenm package in R 4.4.1 (Cobos et al., 2019). The model consists of five feature types: linear (L), quadratic (Q), hinge (H), product (P), and threshold (T) (Wan et al., 2020). When these features were combined, 31 feature combinations were generated, with the default setting being FC = LQPH. The regularization multiplier was set within the range of 0.1 to 4, with an interval of 0.5, resulting in a total of 8 different regularization multiplier values. Using the Kuenm package, a total of 248 parameter combinations (31 feature combinations × 8 regularization multipliers) were tested, with 75% of the data utilized as the training set. Model performance was compared based on the receiver operating characteristic (ROC) curve, omission rate, and AICc (Akaike Information Criterion corrected) values to identify the optimal model. The parameters obtained from this optimal model were then applied to MaxEnt to establish the final model.

AUC (Area Under the Curve) refers to the area under the ROC curve and is commonly used to assess model accuracy (Parodi et al., 2022). It is not influenced by the proportion of subjects in the analysis sample. AUC values range from 0 to 1, with higher values indicating better model fit, higher accuracy, and greater reliability. AUC values between 0.5 and 0.6 suggest model failure, between 0.6 and 0.7 indicate poor performance, between 0.7 and 0.8 represent fair performance, between 0.8 and 0.9 suggest good performance, and values between 0.9 and 1 indicate excellent performance. In this study, AUC values were used to assess the predictive effectiveness of the models. Larger AUC values indicate a stronger correlation between the modeled geographic distribution of *V. officinalis* and environmental factors, suggesting that the model’s predictive performance is better (Ma et al., 2021).

### 2.4 Data processing for the execution of the MaxEnt model

To further investigate the changes in the suitable habitat area of *V. officinalis* under current and various future scenarios, ArcGIS 10.8.1 software was used to delineate and visualize the species’ suitable habitat. The Maximum Test Sensitivity Plus Specificity (MTSPS) threshold was chosen for habitat delineation, as it combines the sensitivity and specificity of the model to classify suitable areas (Liu et al., 2005). The values derived directly from the MaxEnt model were used for this classification. The average result of the MaxEnt model in ASC format was imported into ArcGIS, and the Reclassification tool was employed to categorize the suitable areas into non-suitable areas (0–MTSPS), low-suitability areas (MTSPS–0.5), medium-suitability areas (0.5–0.7), and high-suitability areas (0.7–1). The distribution area of the suitable habitats was then calculated by determining the number of grids in each suitability class.

In ArcGIS, the current and future suitable habitats of *V. officinalis* were classified into two categories: unsuitable habitats (0 to MTSPS) and suitable habitats (MTSPS to 1). The “Intersect” function in ArcGIS was then used to overlay the current suitable habitat with the future suitable habitat distribution. Based on this analysis, the future suitable habitat distribution of *V. officinalis* was defined as expansion areas, contraction areas, and retention areas. The geometric centroid of the suitable habitat was defined as the central point of the habitat’s distribution. The location of this centroid represents the overall spatial position of the suitable habitat. Under the assumption that *V. officinalis* has migration capabilities and ignoring interspecific interactions and other natural factors, the geometric centroid for each scenario was calculated using ArcGIS’s zonal geometry statistics tool (Lenoir et al., 2008). This allowed for the creation of a vector file representing the direction and magnitude of centroid movement between adjacent periods, illustrating the trend and distance of centroid migration (Li et al., 2019).

## 3 Results

### 3.1 Model optimization and accuracy evaluation

**Figure 2:**
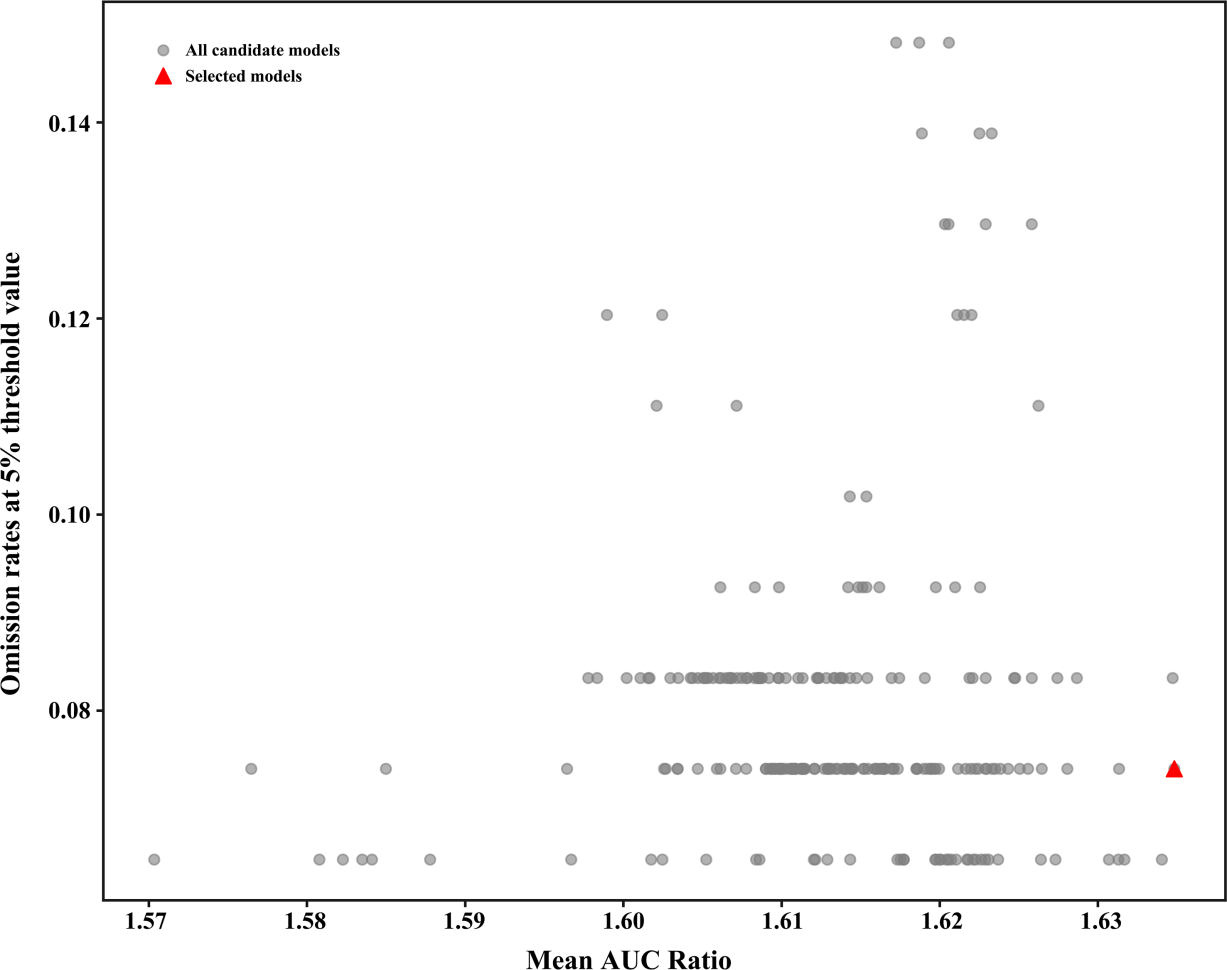
MaxEnt model parameter optimization results.

In the MaxEnt model, the Mean AUC Ratio represents the model’s predictive ability relative to random prediction. A higher value indicates better predictive performance of the model. When the default parameters FC = LQPH and RM = 1 are used, the Mean AUC Ratio is 1.6281. After optimizing the model using the Kuenm package and selecting the parameters FC = LQPT and RM = 1, the Mean AUC Ratio increases to 1.6348. This value is significantly higher than the result obtained with the default parameters. Additionally, the optimized model has a lower AICc value. A lower AICc indicates better model fit and lower complexity, suggesting that the optimized model offers better interpretability. Therefore, in this study, FC = LQPT and RM = 1 were chosen as the parameter settings for the final model of *V. officinalis* distribution (**Figure 2**). After running both the initial and final models, it was found that under the optimized parameters, the AUC value of the MaxEnt model increased from 0.920 in the initial model to 0.934, indicating a more accurate model prediction (**Table 2**).

**Table 2:** Performance evaluation of MaxEnt model under initial and optimized parameters.

Model evaluation	Feature combination	Regularization multiplier	Mean AUC Ratio	AUC value
Default	LQPH	1	1.6281	0.920
Optimized	LQPT	1	1.6348	0.934

### 3.2 The influence of key environmental variables on the distribution of *V. officinalis*

**Figure 3:**
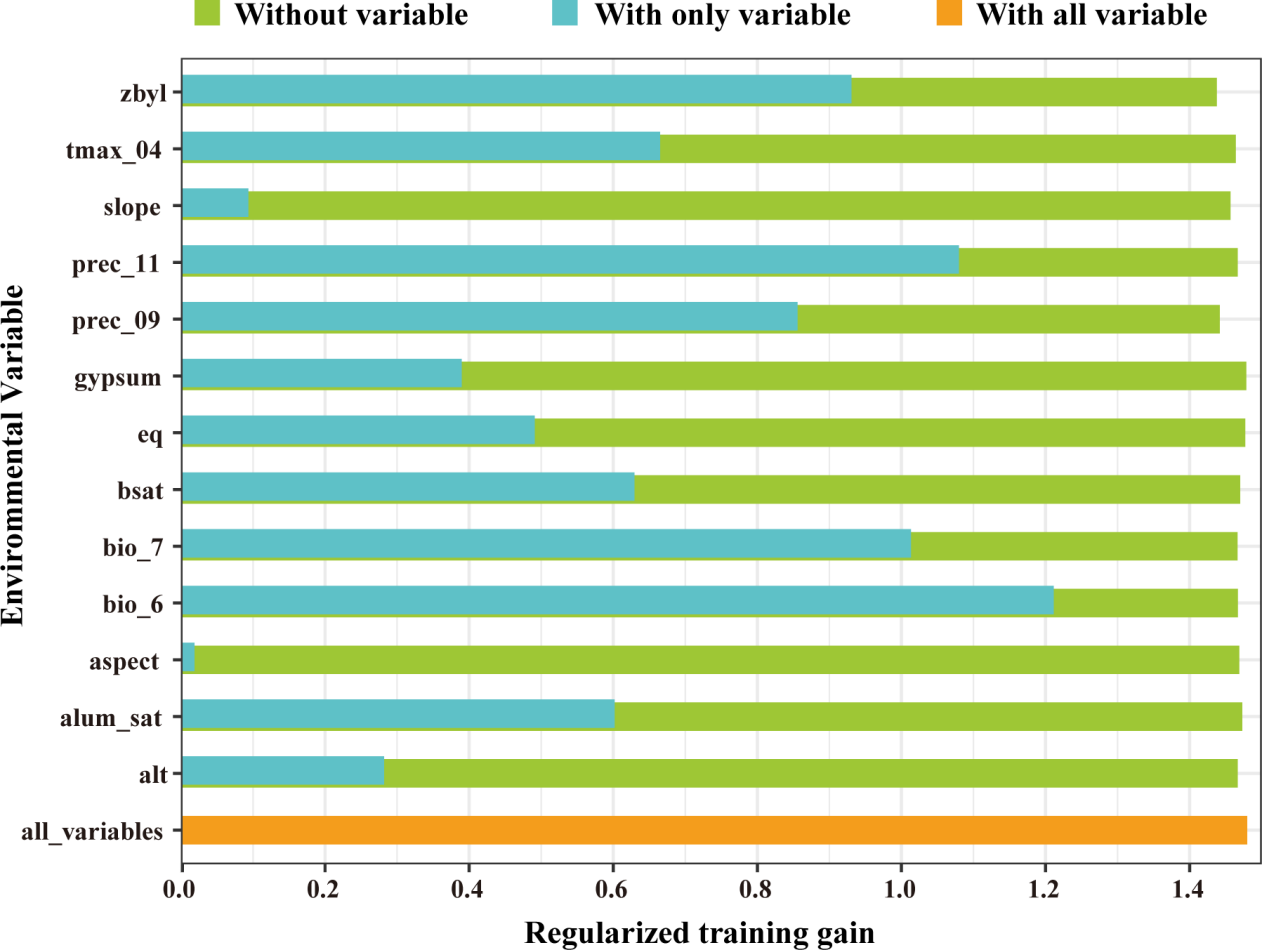
Results of jackknife test for the importance of the variables for MaxEnt.

We analyzed the influence of 13 key environmental variables on the distribution of *V. officinalis* using the MaxEnt model. Based on their contribution rates, bio_6 (Min Temperature of Coldest Month) and bio_7 (Temperature Annual Range) were identified as the major factors for model construction, with a cumulative contribution rate of 81.6%. Among these, bio_6 contributed 72.8%. The environmental variables with relatively smaller contributions included zbyl (5.1%), prec_09 (4.6%), slope (4.3%), prec_11 (3%), alt (2.5%), tmax_04 (2.3%), eq (1.4%), bsat (0.9%), alum_sat (5.1%), aspect (5.1%), and gypsum (5.1%), which together accounted for 18.4% (**Table 3**). Additionally, we assessed the importance of the environmental variables using the Jackknife method based on the generated results. The Jackknife test indicated that when running the model with individual environmental variables, the highest regularized training gain values were achieved by bio_6, bio_7, and prec_11 (**Figure 3**). Therefore, Min Temperature of Coldest Month, November precipitation, and Temperature Annual Range are considered the primary environmental variables influencing the suitable distribution of *V. officinalis*.

**Table 3:** The contribution rate of environmental variables.

Variable code	Environmental factor	Unit	Percent contribution/%	Permutation importance/%
bio_6	Min Temperature of Coldest Month	°C	72.8	47.3
bio_7	Temperature Annual Range	°C	8.8	10.5
zbyl	Vegetation Classification		3.2	7.1
prec_09	Precipitation in September	mm	2.9	5.8
slope	Slope	°	2.7	4.4
prec_11	Precipitation in November	mm	2	4
alt	Altitude	m	1.6	2.2
tmax_04	Maximum Temperature in April	°C	1.5	6.3
eq	Calcium Carbonate	%	1.1	5.6
bsat	Base Saturation	%	1.1	2.4
alum_sat	Aluminium saturation	%	1	1.8
aspect	Aspect	rad	0.8	1.4
gypsum	Gypsum content	%	0.3	1.2

**Figure 4:**
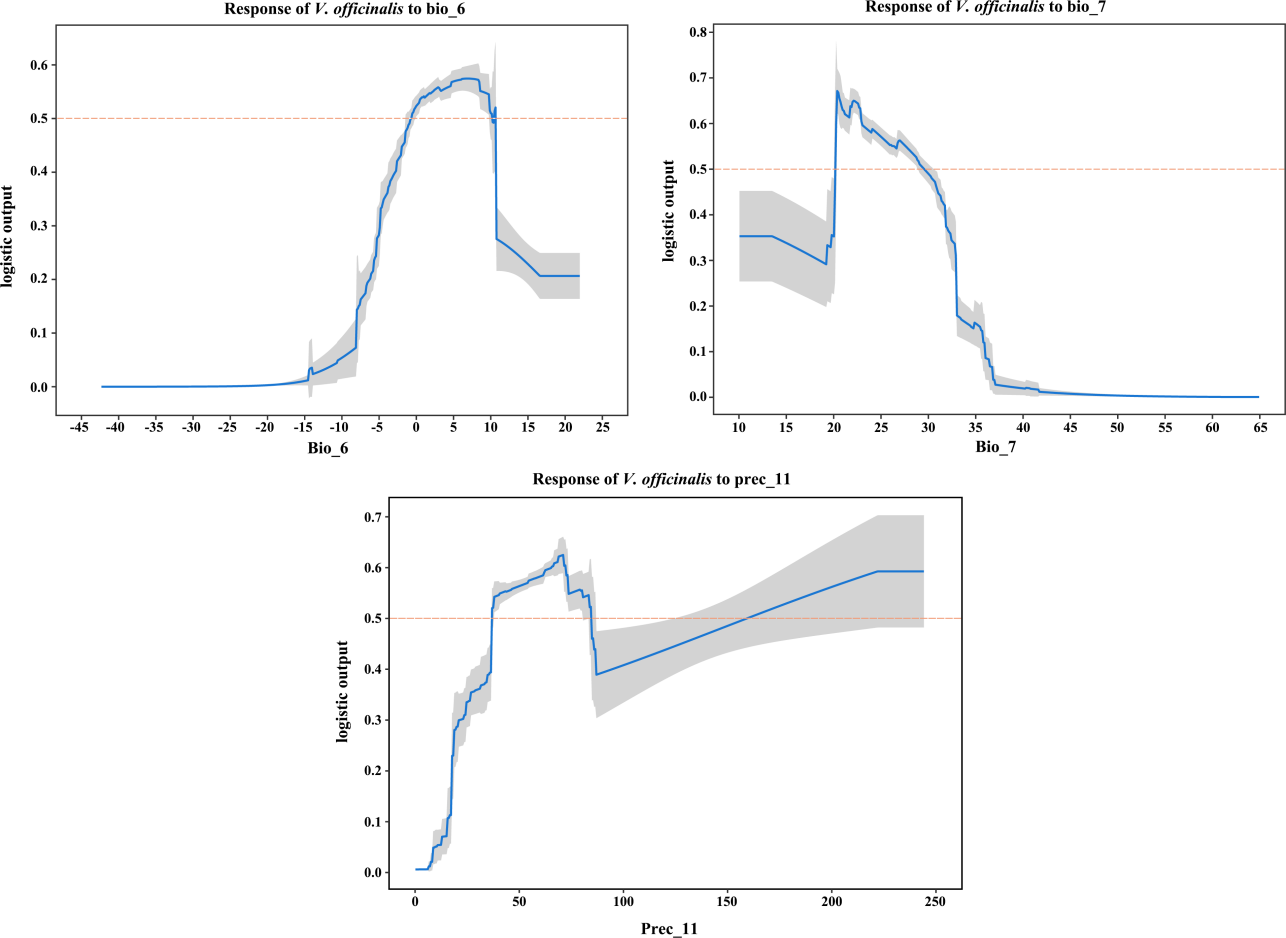
Response curves of key environmental variables (bio_6, bio_7, prec_11) influencing the suitable distribution of *V. officinalis*.

To visualize the response curves for the key environmental variables, we selected the variables for which the logistic output value exceeds 0.5, indicating that the corresponding environmental factor values are conducive to plant growth. The most favorable conditions for *V. officinalis* survival were observed when Min Temperature of Coldest Month ranged from -0.66°C to 10.20°C, Temperature Annual Range ranged from 20.20°C to 29.54°C, and November precipitation ranged from 36.94 mm to 84.36 mm or from 159.48 mm to 224.2 mm (**Figure 4**).

### 3.3 The suitable distribution of *V. officinalis* under current climatic conditions

**Figure 5:**
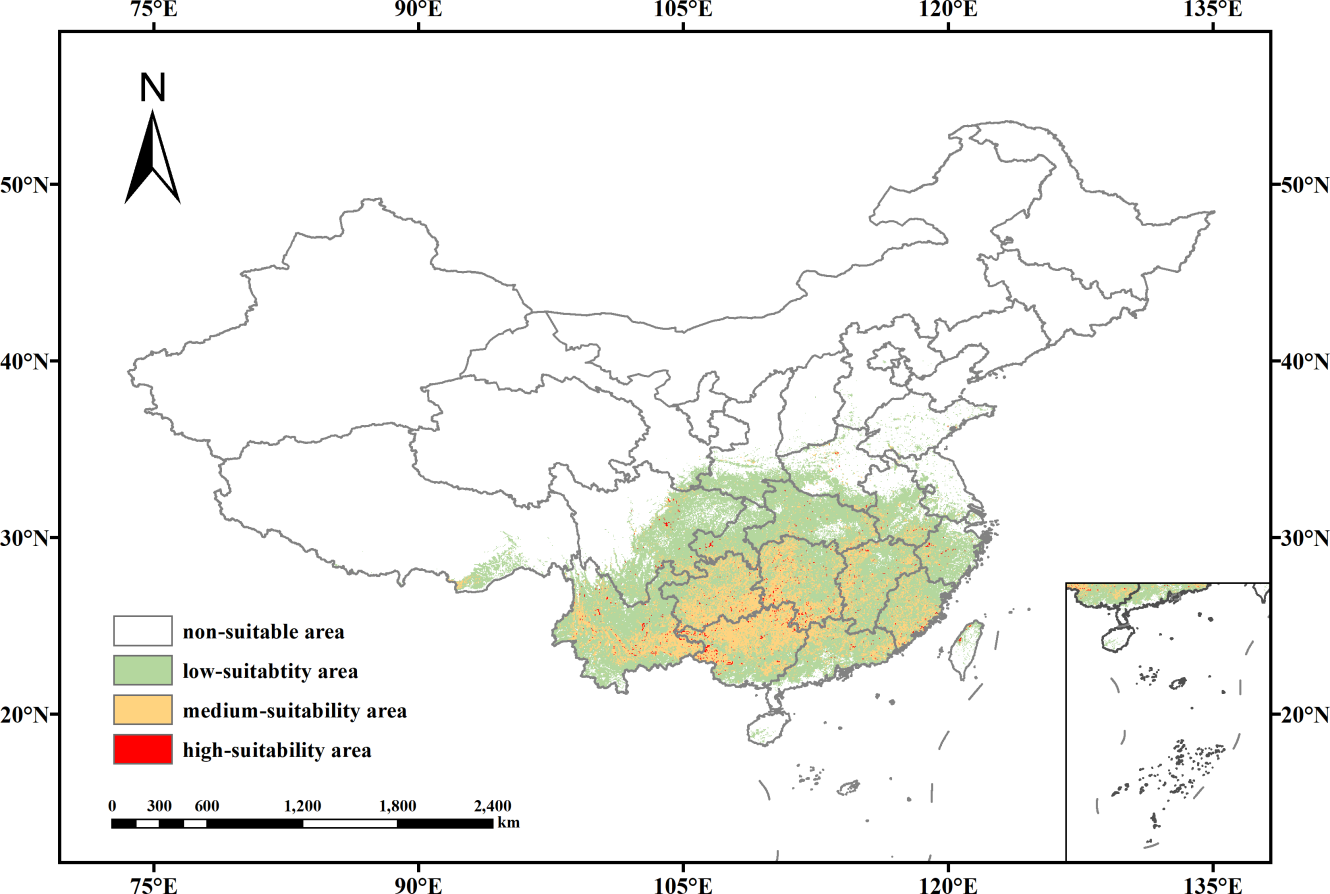
Potential suitable distribution of *V. officinali*s under current climatic conditions.

Based on the results of the MaxEnt model, the potential suitable habitats for *V. officinalis* have been classified into four categories: unsuitable areas (0–0.2037), low suitability areas (0.2037–0.5), medium suitability areas (0.5–0.7), and high suitability areas (0.7–1) (**Figure 5**). Under current climatic conditions, the total suitable habitat area for *V. officinalis* in China is estimated to be 2.06 × 10^6^ km^2^, accounting for 21.39% of the country’s total land area, primarily distributed in Central China, East China, and South China. The high suitability areas are mainly concentrated in the eastern and southeastern regions, including economically developed areas such as the Yangtze River Delta and the Pearl River Delta, covering an area of 2.49 × 10^4^ km^2^, which represents 0.23% of the total land area of China. The medium suitability areas are more widely distributed, particularly in the central region, with significant coverage in provinces such as Henan, Hubei, and Hunan in Central China. Additionally, there are distributions in certain provinces in North and Southwest China, such as Sichuan and Chongqing, covering an area of 5.44 × 10^5^ km^2^, which accounts for 5.61% of the total land area of China. The low suitability areas are primarily found in the central and eastern parts of China, including the middle and lower reaches of the Yangtze River plain and parts of the southeastern coastal regions. These areas encompass several provinces, including Jiangsu, Zhejiang, Anhui, Jiangxi, and Fujian, covering an area of 1.49 × 10^6^ km^2^, which accounts for 15.51% of the total area of China.

### 3.4 The suitable distribution of *V. officinalis* under future climate conditions

**Figure 6:**
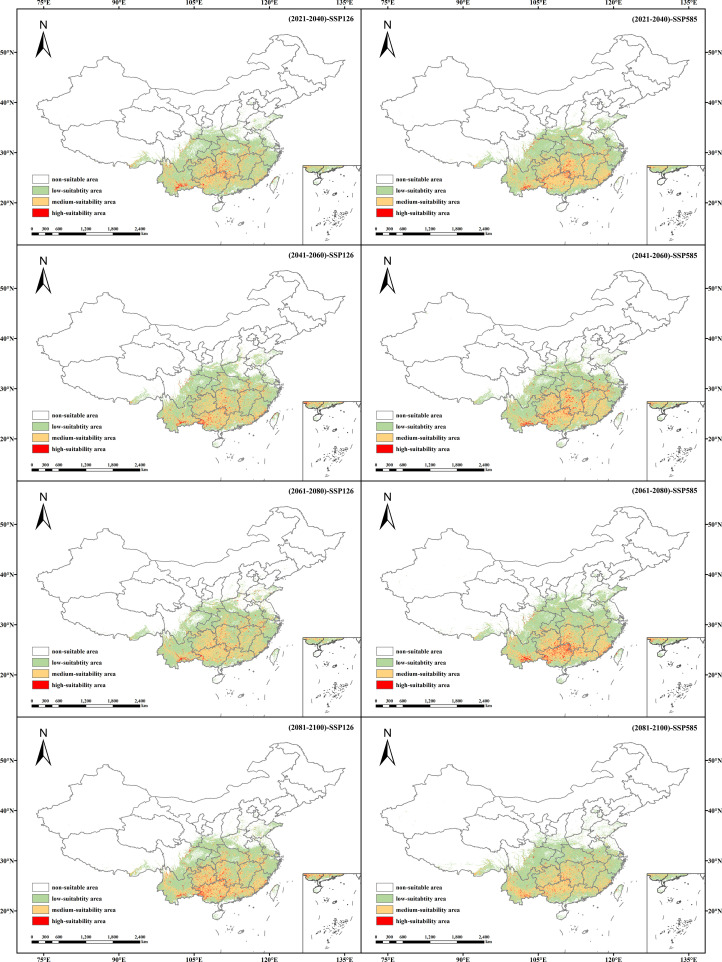
Suitable habitat distribution of *V. officinalis* under different future climate scenarios (SSP1-2.6 and SSP5-8.5).

Based on the predictions made by MaxEnt for two emission models across four future periods—2020 to 2040, 2041 to 2060, 2061 to 2080, and 2081 to 2100—the distribution of future suitable areas for *V. officinalis* in China and the areas classified into different suitability growth grades using the MTSPS method were determined (**Table 4**; **Figure 6**).

**Table 4:** Area of suitable habitats for *V. officinalis* under current and future climate scenarios by suitability levels.

Decade scenarios	Predicted area (× 10^4^ km^2^)				
	Low habitat suitability	Medium habitat suitability	High habitat suitability	Unsuitable habitat	Total suitable area
Current	149.25	54.44	2.19	756.47	205.88
2030s-SSP1-2.6	146.45	67.12	5.24	743.55	218.81
2050s-SSP1-2.6	140.59	71.61	5.40	744.74	217.61
2070s-SSP1-2.6	141.71	72.74	4.29	743.62	218.74
2090s-SSP1-2.6	131.40	81.74	8.03	741.18	221.17
2030s-SSP5-8.5	139.26	86.45	5.68	730.96	231.39
2050s-SSP5-8.5	133.88	81.65	7.69	739.13	223.22
2070s-SSP5-8.5	151.54	82.19	8.15	720.47	241.89
2090s-SSP5-8.5	146.85	73.48	3.54	738.48	223.87

Under the SSP1-2.6 scenario, the total suitable area for *V. officinalis* is projected to show an increasing trend compared to current climate conditions. By the 2090s, the total suitable area is expected to increase by 1.53 × 10^5^ km^2^, with high-suitability areas expanding by 5.84 × 10^4^ km^2^ and medium-suitability areas increasing by 2.73 × 10^5^ km^2^. However, by the 2090s, the area of low suitability is expected to continue declining, with a reduction of 1.79 × 10^5^ km^2^.

Under the SSP5-8.5 scenario, the total suitable area for *V. officinalis* also shows an increasing trend. By the 2070s, the total suitable area is expected to reach its maximum value of 2.42 × 10^6^ km^2^, representing an increase of 3.6 × 10^5^ km^2^ compared to the current climate. The area of high suitability shows the most significant increase, with an expansion of 5.96 × 10^4^ km^2^. The area of medium suitability is projected to increase in all future decades, although the rate of increase is expected to gradually decrease over time. The area of low suitability, similar to the SSP1-2.6 scenario, is expected to show an overall decreasing trend.

### 3.5 Spatial pattern changes in the future potential distribution of *V. officinalis*

**Figure 7:**
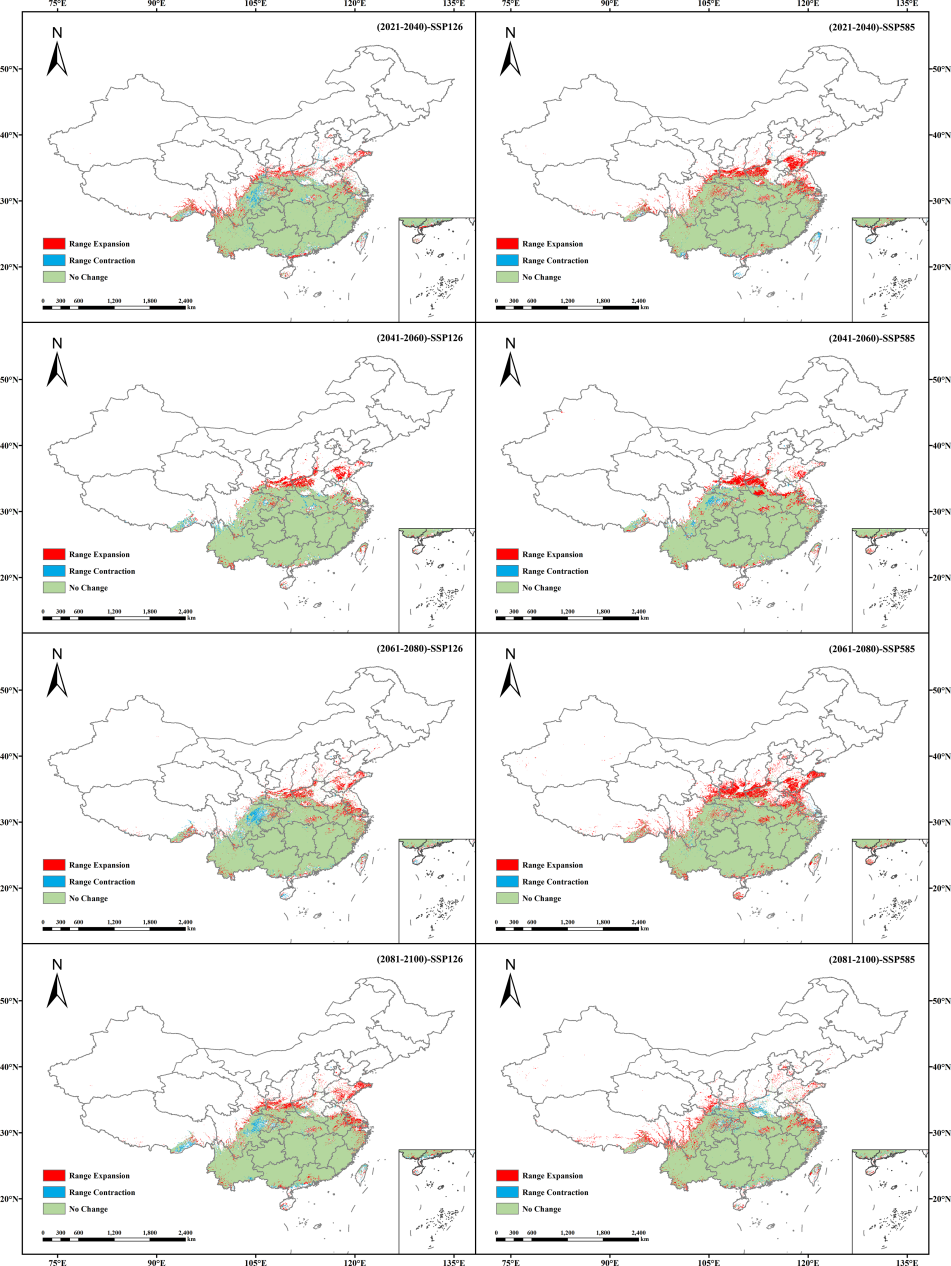
Spatial pattern changes in the potential suitable habitats of *V. officinalis* under different future climate scenarios.

**Figure 8:**
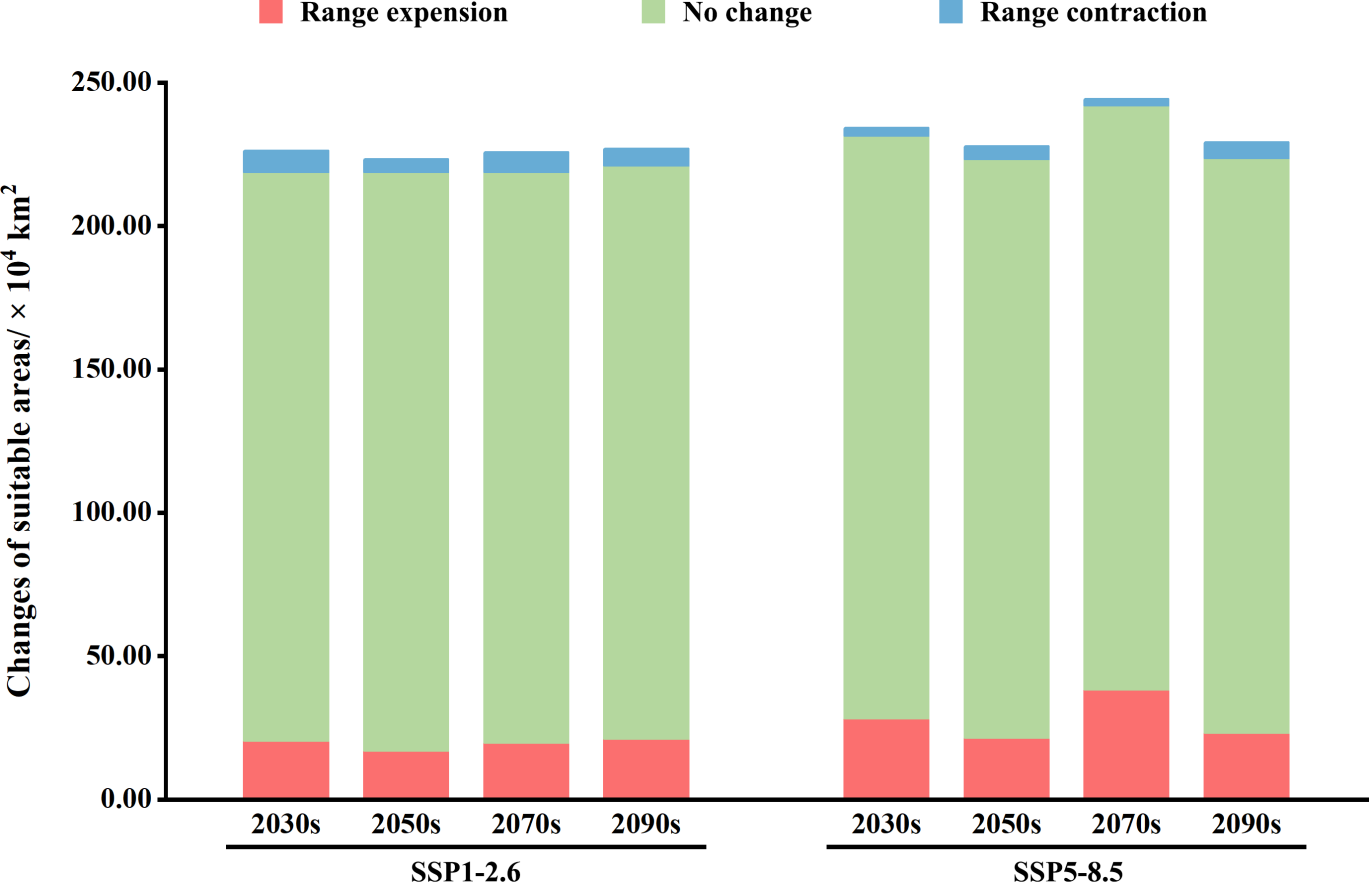
Stacked chart of spatial pattern changes in the potential suitable habitats of *V. officinalis* under different future climate scenarios.

Under eight future climate scenarios, the potential distribution of *V. officinalis* suitable habitats was compared with its current distribution (**Figures 7**, **8**). Results indicate that under the SSP1-2.6 scenario, the potential suitable habitat of *V. officinalis* changes relatively gradually, with expansion areas primarily concentrated around the periphery of existing habitats, especially in northern and higher-altitude regions. The expanded area increases from 2.04 × 10^5^ km^2^ in the 2030s to 2.1 × 10^5^ km^2^ in the 2090s, with overall minor variation. The stable areas, where no changes occur, remain dominant, ranging between 1.98 × 10^6^ and 2.02 × 10^6^ km^2^. In contrast, areas of habitat reduction show slight fluctuations, decreasing from 7.44 × 10^4^ km^2^ in the 2030s to 4.59 × 10^4^ km^2^ in the 2050s, with a minor rebound in the 2070s and 2090s. The reduction areas are mainly located in eastern Sichuan, Hubei, and southeastern Tibet. Overall, the expansion of suitable habitats predominantly occurs around existing distributions, with the centroid shifting slightly northward and the spatial pattern remaining relatively stable.

In contrast, under the SSP5-8.5 scenario, *V. officinalis* experiences a substantial expansion in its potential suitable habitats, with new areas extending significantly toward northern high-latitude regions. The expanded area increases rapidly from 2.82 × 10^5^ km^2^ in the 2030s to a peak of 3.82 × 10^5^ km^2^ in the 2070s. Stable areas (unchanged suitable habitats) remain relatively consistent, ranging from 2.01 × 10^6^ to 2.03 × 10^6^ km^2^, with only minor fluctuations. Meanwhile, the reduced habitat area occupies a smaller proportion overall and shows a declining trend, decreasing from 2.75 × 10^4^ km^2^ in the 2030s to 2.26 × 10^4^ km^2^ in the 2070s, followed by a slight rebound to 5.45 × 10^4^ km^2^ in the 2090s. The reduction areas are primarily concentrated in Sichuan, Shaanxi, and Henan provinces. Spatially, the distribution of suitable habitats under this scenario exhibits a pronounced northward shift, with significant expansion, particularly in high-latitude regions, reflecting a strong adaptive expansion pattern in these areas.

### 3.6 Centroid migration of suitable habitats in future periods

**Figure 9:**
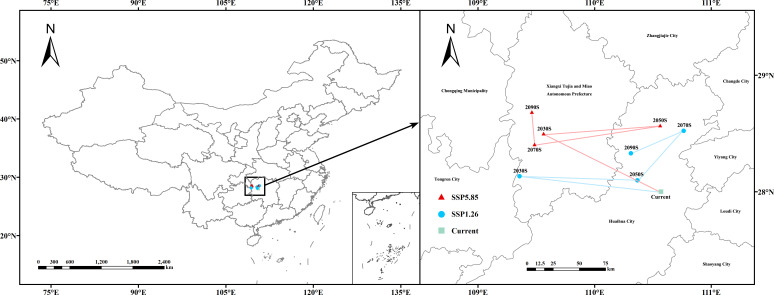
Migration trajectory of the geometric centroid of suitable areas for *V. officinalis* under future climate scenarios.

Using the MTSPS value (MTSPS = 0.2037) as the threshold, the suitable and unsuitable areas for *V. officinalis* were classified. The migration of the centroid of the suitable area over time under two emission scenarios, SSP1-2.6 and SSP5-8.5, was analyzed using ArcGIS, and the centroid migration trajectory was plotted (**Figure 9**).

Under the current climate, the centroid of the suitable area for *V. officinalis* is located in Shupu County, Huaihua City, Hunan Province. Under the SSP1-2.6 emission scenario, from 2021 to 2040, the centroid migrates 136.98 km northwest to Fenghuang County, Xiangxi Tujia and Miao Autonomous Prefecture, Hunan Province; from 2041 to 2060, it moves 111.66 km southeast to Chenxi County, Huaihua City, Hunan Province; from 2061 to 2080, the centroid shifts 69.84 km northeast to Yuanling County, Huaihua City, Hunan Province; and from 2081 to 2100, the centroid continues to migrate 55.60 km southwest within Yuanling County, Huaihua City, Hunan Province.

Under the SSP5-8.5 emission scenario, from 2021 to 2040, the centroid migrates 128.42 km northwest to Baojing County, Xiangxi Tujia and Miao Autonomous Prefecture, Hunan Province; from 2041 to 2060, it shifts 111.61 km northeast to Yuanling County, Huaihua City, Hunan Province; from 2061 to 2080, the centroid moves 121.58 km southwest to Huayuan County, Xiangxi Tujia and Miao Autonomous Prefecture, Hunan Province; and from 2081 to 2100, the centroid migrates 35.06 km northwest back to Baojing County, Xiangxi Tujia and Miao Autonomous Prefecture, Hunan Province.

## 4 Discussion

Ecological niche models predict the potential distribution of species based on certain algorithms that estimate their ecological requirements. Therefore, improving the accuracy of model predictions is crucial for the construction of ecological niche models (Seo et al., 2021). To accurately predict the suitable habitat of *V. officinalis*, this study optimized the MaxEnt model from several aspects, including species distribution points, selection of environmental variables, and model parameter settings, to enhance the prediction accuracy. First, the distribution data of *V. officinalis* were screened using the ENMTools software to exclude data with high spatial autocorrelation, thereby reducing sampling bias. Second, this study collected 90 environmental factors, including climate, soil, and topography, to provide a more comprehensive prediction of the suitable habitat for *V. officinalis*. A correlation analysis was performed, and environmental variables with an absolute correlation greater than 0.8 were removed to avoid multicollinearity, which could lead to model overfitting. Finally, the Kuenm package was used to optimize the feature combinations and tuning of the MaxEnt model, which not only mitigated overfitting but also improved the model’s prediction accuracy and reliability. The optimized model achieved an AUC value of 0.934, indicating that the model’s predictions are highly reliable.

Research has shown that the growth and distribution of *V. officinalis* are significantly influenced by climatic factors, with temperature being particularly crucial. The plant can survive completely when winter temperatures are above -4°C, but it dies entirely when temperatures fall below -17°C. Flowering requires temperatures above 16°C, and seed germination necessitates a daily average temperature exceeding 14°C, with daytime temperatures needing to be above 19°C (Woodward, 1997). Suitable temperatures are also important for the synthesis of secondary metabolites in *V. officinalis*, as both extreme high and low temperatures can inhibit the accumulation of its chemical components (Pant et al., 2021). The results of the MaxEnt model in this study also corroborate these findings. When the lowest temperature of the coldest month is in the range of -0.66 to 10.20°C, annual temperature range is between 20.20 and 29.54°C, and November precipitation falls within the range of 36.94–84.36 mm or 159.48–224.2 mm, the conditions are most suitable for *V. officinalis* survival. Notably, the lowest temperature of the coldest month contributed 72.8% to the model’s prediction of suitable habitats. These findings highlight that warm summer conditions are favorable for the reproductive success of *V. officinalis*, while extreme cold winters may lead to substantial mortality. Although snow cover can provide insulation under severe cold conditions, low temperatures remain a key limiting factor for its distribution. Based on the above research results, the artificial cultivation of *V. officinalis* should take into account the suitability of temperature and precipitation, particularly avoiding winter temperatures below -4°C and extreme high temperatures. To ensure stable growth and high-quality secondary metabolite synthesis, greenhouse cultivation or the selection of regions with moderate climate and adequate precipitation is recommended. Additionally, proper irrigation and temperature control will help promote healthy growth and effective distribution of *V. officinalis.*

Currently, *V. officinalis* is found in provinces south of the Qinling Mountains and in Xinjiang, with the main production areas located in Hunan, Guizhou, and other regions. It is also found in Anhui, Zhejiang, Henan, Jiangxi, Fujian, Hebei, Sichuan, and other provinces. The results of this study also confirm that, under the current climatic scenario, *V. officinalis* is primarily distributed in the Central, Eastern, and Southern regions of China. The total suitable habitat area in China is 2.06×10^6^ km^2^, accounting for 21.39% of the country’s total land area. Therefore, the model’s predicted potential suitable habitats align well with the actual distribution. Furthermore, the MTSPS method used in this study for delineating the suitable habitat of *V. officinalis* combines both the model’s sensitivity and specificity. In contrast, the commonly used natural distribution breakpoint method relies solely on computational results, lacking integration with real-world variability (Liu et al., 2005). The MTSPS approach is widely adopted in many studies to delineate species distributions (Zhang et al., 2018; Mahatara et al., 2021).

Under future climate conditions, the suitable habitat area for *V. officinalis* is projected to generally increase, with the centroid of the suitable habitat shifting toward higher latitudes. As the impact of future climate change progresses, the spatial shifts in the suitable habitat of *V. officinalis* are largely consistent with the movement of the centroid, reflecting a trend of northward expansion. This result is in line with findings from related studies. For example, under climate change, the future suitable habitats of plants such as *Astragalus membranaceus* and *Panax notoginseng* in China are gradually migrating toward higher latitudes (Zhan et al., 2022; Wen et al., 2024). Studies have shown that global warming will drive species migration to higher latitudes or elevations (Changjun et al., 2021). In the context of global climate warming, the annual increase in precipitation and temperature will further enhance the suitability of habitats for *V. officinalis* (Solomon et al., 2007). Additionally, under future climate scenarios, northern China, influenced by geographical factors, temperature changes, and greenhouse gas emissions, is expected to experience significant variations in precipitation and annual average temperature. These increases will be much more pronounced than in southern China, leading to a trend of *V. officinalis* migrating toward higher latitudes (Yang et al., 2021).

Despite the overall trend of expansion towards higher latitudes, the geometric center of the suitable habitat for *V. officinalis* does not continuously migrate northward under future climate scenarios. Instead, it exhibits periodic fluctuations within Hunan Province. For example, under the SSP1-2.6 scenario, the migration trajectory of the center is “northwest → southeast → northeast → southwest,” while under the SSP5-8.5 scenario, it follows the path “northwest → northeast → southwest → northwest.” The reasons for this fluctuation may involve the synergistic effects of multiple factors: (1) Non-climatic factors, such as topography and vegetation types, regulate the local expansion direction through microhabitat heterogeneity. For instance, increased precipitation in the future Wuling Mountains in western Hunan could create moist valley habitats that promote westward migration; (2) Phase changes in climatic factors under different emission scenarios, such as the SSP1-2.6 scenario, where the warming in winter temperatures in the middle and lower reaches of the Yangtze River slows, thus inhibiting northern range expansion; (3) Boundary effects due to the model’s response to extreme climate thresholds, which may lead to uncertainty in high-latitude predictions. This finding suggests that species distribution under climate change is a result of the nonlinear coupling of climate, topography, and vegetation. Therefore, during the development of *V. officinalis* resources, special attention should be given to the ecological connectivity of transition zone habitats (such as the Wuling Mountains) to mitigate the fragmentation risk of distribution caused by climate fluctuations.

However, it is important to note that while the MaxEnt model used in this study to predict the suitable habitat of *V. officinalis* in China yielded highly accurate results (AUC value of 0.934), these predictions remain theoretical estimates, and the actual suitable habitats may be influenced by more complex factors. First, although we selected 90 environmental variables to construct the model, these variables cannot comprehensively replace all potential factors that may affect species distribution. For example, factors such as economic development, land-use changes, government policies, and human disturbances could also significantly impact the actual distribution of *V. officinalis*. Therefore, future research should consider a broader range of environmental data and socio-economic factors to enhance the applicability and accuracy of model predictions. Secondly, the uncertainty in predicting species distribution under climate change is also a concern, particularly in long-term climate scenarios, where actual changes may deviate from model assumptions. To further optimize prediction results and improve the model’s reliability, future studies should collect more detailed and diverse data, and continually update models to account for the evolving environmental conditions.

## 5 Conclusion

This study systematically predicted and analyzed the potential distribution of *V. officinalis* in China under current and future climate conditions using an optimized MaxEnt model. The results indicate that the growth of *V. officinalis* is significantly influenced by temperature and precipitation, with the minimum temperature of the coldest month (bio_6) being the most critical factor, contributing 72.8% to the distribution of suitable habitats.

Under current climate conditions, the suitable habitats for *V. officinalis* are primarily concentrated in Central China, East China, and South China, covering a total area of 2.06 × 10^6^ km^2^, which accounts for 21.39% of China’s land area. Under future climate scenarios (SSP1-2.6 and SSP5-8.5), the total area of suitable habitats shows an overall increasing trend, with the most significant expansion projected to occur by the 2070s under the high-emission scenario (SSP5-8.5). Furthermore, future climate change is expected to shift the centroid of suitable habitats progressively northward, with high-latitude and high-altitude regions demonstrating considerable potential for expansion.

By optimizing the model parameters, the prediction accuracy was significantly improved, achieving an AUC value of 0.934. This study provides a robust scientific basis for the conservation of *V. officinalis* resources, cultivation planning, and the development of sustainable strategies to address climate change. Additionally, the northward expansion of suitable habitats offers critical insights for future introduction and cultivation in northern regions.

## Data Availability

The original contributions presented in the study are included in the article/supplementary material. Further inquiries can be directed to the corresponding author/s.
